# Contrast gain control and horizontal interactions in V1: A DCM study

**DOI:** 10.1016/j.neuroimage.2014.01.047

**Published:** 2014-05-15

**Authors:** D.A. Pinotsis, N. Brunet, A. Bastos, C.A. Bosman, V. Litvak, P. Fries, K.J. Friston

**Affiliations:** a The Wellcome Trust Centre for Neuroimaging, University College London, Queen Square, London WC1N 3BG, UK; b Ernst Strüngmann Institute (ESI) for Neuroscience in Cooperation with Max Planck Society, Deutschordenstraße 46, 60528 Frankfurt, Germany; c Donders Institute for Brain, Cognition, and Behaviour, Radboud University Nijmegen, 6525 EN Nijmegen, Netherlands; d Cognitive and Systems Neuroscience Group, Swammerdam Institute for Life Sciences, Center for Neuroscience, University of Amsterdam, 1098 XH Amsterdam, Netherlands; e Department of Neurological Surgery, University of Pittsburgh, PA 15213, USA; f Center for Neuroscience and Center for Mind and Brain, University of California-Davis, Davis, CA 95618, USA

**Keywords:** Neural field theory, Dynamic causal modelling, Contrast, Attention, Connectivity, Gamma oscillations, V1, Electrocorticography, Visual cortex, Electrophysiology

## Abstract

Using high-density electrocorticographic recordings – from awake-behaving monkeys – and dynamic causal modelling, we characterised contrast dependent gain control in visual cortex, in terms of synaptic rate constants and intrinsic connectivity. Specifically, we used neural field models to quantify the balance of excitatory and inhibitory influences; both in terms of the strength and spatial dispersion of horizontal intrinsic connections. Our results allow us to infer that increasing contrast increases the sensitivity or gain of superficial pyramidal cells to inputs from spiny stellate populations. Furthermore, changes in the effective spatial extent of horizontal coupling nuance the spatiotemporal filtering properties of cortical laminae in V1 — effectively preserving higher spatial frequencies. These results are consistent with recent non-invasive human studies of contrast dependent changes in the gain of pyramidal cells elaborating forward connections — studies designed to test specific hypotheses about precision and gain control based on predictive coding. Furthermore, they are consistent with established results showing that the receptive fields of V1 units shrink with increasing visual contrast.

## Highlights

• A new observation model suitable for ECoG recordings.
• An canonical microcircuit field model.
• A DCM treatment of multiple experimental conditions and trial-specific effects.
• Excitation-inhibition balance in terms of strength and dispersion.

## Introduction

This paper models high-density electrocorticographic (ECoG) multi-electrode data with neural fields to characterise local cortical excitability (the kinetics of postsynaptic potentials) and microstructure (the spatial summation of receptive fields). It is well-known that the local processing of information in the visual cortex is highly sensitive to the contrast of the stimuli being processed; see e.g. Sceniak et al. (2001, 2006). Crucially, single unit studies have demonstrated interactions between the contrast and spatial extent of stimuli that change the form of classical and non-classical receptive fields. This suggests that contrast manipulations induce changes in effective horizontal interactions in the striate cortex (Nauhaus et al., 2008). These changes speak to the nonlinear and locally distributed neuronal computations underlying visually induced responses. The aim of this work was to disambiguate three competing explanations for these contrast effects in terms of changes in the postsynaptic gain of neuronal populations, the strength of intrinsic (interlaminar) connectivity or changes in the effects of horizontal connections.

Our work exploits information in high density – spatially resolved – electrophysiological data to characterise contrast dependent neuronal mechanisms that underlie early visual processing. We used data from an electrocorticographic (ECoG) multi-electrode array acquired whilst a monkey fixated a central point and was stimulated with visual gratings of varying contrast. Our focus was on the three way relationship between gamma responses induced by stimuli of varying contrast, the implicit spatial summation over V1 receptive fields (RFs) and the underlying balance of inhibition and excitation in neuronal interactions. To address this relationship, we used dynamic causal modelling (DCM; Friston et al., 2003) to explain visually induced responses to stimuli of different contrast levels.

DCM has recently been extended to neural fields (Pinotsis et al., 2012). The advantage of neural fields (over neural masses or point sources) is that they can accommodate the spatial or topographic properties of cortical sources. Neural field models include horizontal intrinsic connections within layers or laminae of the cortical sheet. Neural fields have a long and illustrious history in mathematical neuroscience, see Deco et al. (2008) and Nunez (1995) for a review. These models prescribe the time evolution of cell activity – such as mean depolarisation or (average) action potential density – and are often used to *simulate* neural activity. Here, we follow a different route and use them to *fit* observed data. This allows us to estimate synaptic parameters and answer detailed questions about functional architectures.

This paper introduces the following three technical and conceptual advances, in relation to our previously published work on DCM for neural fields (Pinotsis et al., 2012): (i) A new observation model suitable for ECoG recordings. (ii) An extension of the classical Jansen and Rit model (introduced in our earlier work) to a field version of the canonical microcircuit model (Bastos et al., 2012; Pinotsis et al., 2013a). (iii) A treatment of multiple experimental conditions that allows for trial-specific effects on parameter subsets — accounting for the effects of various stimulus properties.

We have previously considered local field potential measurements, where the sensor is close to the cortical source and the lead field resembles a delta function; in other words, the activated cortical patch is large in relation to the area seen by the sensor (Pinotsis et al., 2012). Conversely, we have modelled MEG measurements, where the activated cortical patch is small in relation to the area seen by the sensor (Pinotsis et al., 2013a). In this work, we consider ECoG multi-electrode data, where each sensor sees a different (unknown) part of the cortical patch activated by the visual stimulus. This means the lead fields are functions of the sensor location – relative to the centre of the source – and their spatial dispersion. This dispersion reflects the relative extent of source activity picked up by the sensor (and is distinct from the spatial dispersion of horizontal connections, which is also optimised). In short, our generative (forward) model tries to explain responses from multiple sensors and calls for a more detailed consideration of the lead fields associated with each sensor — and the way that electrodes sample local electromagnetic responses over space. This represents an intriguing inverse problem that can, in principle, be solved by inverting a neural field DCM. In this setting, both the parameters governing the spatial aspects of cortical microcircuitry and the spatial characteristics of the lead fields (i.e., sensitivity profiles) have to be estimated.

In this paper, we extend the neural field model of cortical microrcircuitry to distinguish between superficial and deep pyramidal cell populations. This was motivated by theoretical considerations based on predictive coding and experimental observations of spectral asymmetries across depth electrode measurements. The ensuing model is similar to the model described in Pinotsis et al. (2013a) and includes parameters that differentiate between the spatial extent of different horizontal connections. This model also allows for measured signals to be a mixture of activity of different subpopulations in the source space — as opposed to considering only pyramidal cell activity (Pinotsis et al., 2012).

We have used the resulting model to explain condition-specific changes in model parameters associated with differences in the contrast of visual stimuli. This was achieved by simultaneously fitting all the contrast conditions with the same model, whilst allowing only changes in a small subset of parameters to explain condition-specific effects. The objective of this modelling is to assess the evidence for different models or hypotheses of observed (contrast dependent) responses — and compare the ability of different models to explain observed data using Bayesian model comparison. This allows one to test different hypotheses about the synaptic mechanisms underlying contrast dependent effects. Specifically, we asked whether cross spectral responses to parametrically varying contrast can be explained by:

(i) Changes in the strength of recurrent (self) connections of neuronal populations (superficial and deep pyramidal cells): this explanation is the most parsimonious and assumes that the apparent changes in receptive field size (with contrast) can be explained by changes in the nonlinear response properties of individual populations, without any changes in the intrinsic connections between populations or lamina. In particular, following recent dynamic causal modelling of visually induced responses in the visual hierarchy, we hypothesised that contrast would be interpreted by the brain as an increase in the signal-to-noise (precision) of sensory information that would be reflected in an increase in the postsynaptic gain of superficial pyramidal cells (Feldman and Friston, 2010).
(ii) Changes in strength of intrinsic (horizontal) connections between excitatory and inhibitory populations that constitute a canonical microcircuit: this explanation calls upon contrast dependent changes in the sensitivity of different populations as expressed in terms of changes in the strength of intrinsic connectivity. Here, the explanation for the apparent change in receptive field properties rests on the differential contribution of excitatory and inhibitory connections that mediate spatial summation where – crucially – the extent of horizontal interactions remains the same.
(iii) Changes in the spatial dispersion (extent) of horizontal excitatory and inhibitory connections: this explanation rests upon a contrast dependent effect that is expressed differently in long and short range horizontal connections. In contrast to the first two, this explanation entails a change in the effective spatial extent of lateral interactions; as opposed to a change in their strength under a fixed spatial form.

These hypotheses were cast as dynamic causal models that entailed contrast dependent changes in the strength of recurrent connections, the strength of horizontal connections or the extent of horizontal connections (or combinations thereof). The ability of each model (hypothesis) to explain induced responses was evaluated in terms of their Bayesian model evidence; thereby providing evidence in favour of one mechanism over another. Having established the best model or mechanism, we were then able to relate the parameters of that model quantitatively to predicted spectral responses — in particular changes in the frequency of the peak gamma response with stimulus contrast.

In summary, our analysis is based on a simple model of visual cortex that conforms to established neuroanatomical rules. This model can generate differences in gamma responses that depend upon stimulus conditions that are thought to result from the interaction between local excitation and inhibition, (Brunel and Wang, 2003; Kang et al., 2010; Traub et al., 1997). Model predictions were then used in combination with empirical data to estimate receptive field properties, such as the range of spatial summation and connection strengths within and between excitatory and inhibitory pools of neurons. Our focus is on understanding the link between neuronal architecture of the sources and phenotypic differences in spectral responses. These architectures are informed by some key empirical findings, which we now review briefly:

### Contrast modulates spatial summation of receptive fields and gamma peak frequency

V1 receptive fields are composed of an excitatory centre and an inhibitory surround. For example, Sceniak et al. (1999) recorded single units in V1 when monkeys fixated a point on the screen and were shown patches of drifting gratings at various contrasts and sizes. Using a difference of Gaussians model – to fit the spatial extent of contributions of the excitatory centre and inhibitory surround – they showed that at higher contrasts, the excitatory centre of receptive fields in V1 had a smaller stimulus summation field. The authors found that – at lower contrasts – V1 receptive fields were 2.3 times larger than at higher contrasts. These results suggest that receptive fields are not invariant to stimulus properties. Similarly, Kapadia et al. (1999) recorded from superficial cells in monkey V1 whilst they presented oriented bars of varying lengths. They found that V1 receptive fields were on average about 4 times larger at low contrast compared to high contrast, when they were presented in isolation; and about twice as large when they were presented in the context of a textured background. The authors conclude that the excitatory–inhibitory balance between the classical and non-classical receptive field is not static but can be modulated by stimuli. At high contrast, neurons are strongly inhibited when a stimulus falls outside the classical receptive field and encroaches on the non-classical receptive field. At lower contrast, V1 receptive fields have enhanced spatial summation, indicating that inhibition relative to excitation may be reduced.

Gamma band oscillations (30–100 Hz) in V1 are also sensitive to stimulus properties like contrast (and stimulus size) (Ray and Maunsell, 2010). With increasing contrast, gamma oscillations increase in peak frequency (see also Roberts et al., 2013). In our data – at the lowest contrast with a detectable gamma peak (10%) – the gamma frequency is about 47 Hz; whilst in the highest contrast condition (82%), the peak frequency is at about 57 Hz. Gamma band oscillations have been found to depend on neuronal interactions mediated by both gap junctions and synaptic transmission (Traub et al., 2001). In our model, these effects are modelled collectively by intrinsic connectivity constants. In the context of dynamic causal modelling, these quantities have been shown to accurately reflect neurotransmitter density and synaptic efficacy (Moran et al., 2008). A more biologically realistic description of neuronal interactions (that considered neuromodulatory and other conductance-specific mechanisms — and even gap junctions) would entail the use of more elaborate (e.g., conductance based) models at the cost of increased computational demands — in relation to the convolution model used here. Examples of more realistic dynamic causal modelling in this context can be found in Marreiros et al. (2010) and Pinotsis et al. (2013b). Generally speaking, the level of biological detail or complexity that can be entertained rests upon the amount of information in the data. In this provisional (proof of principle) study, we use relatively simple models of canonical microcircuitry that we know can be inverted efficiently. However, one might anticipate subsequent modelling with more realistic models and thereby more refined biophysical hypotheses.

In summary, several experimental studies show that gamma peak frequency, stimulus contrast and the excitatory–inhibitory balance between the classical and non-classical receptive fields are all interconnected. Below, we will address these relationships using Bayesian model comparison of dynamic causal models that embody different hypotheses about contrast-specific changes in the connectivity architectures that underlie receptive fields and induced responses.

## Material and methods

### Experimental protocol

**Fig. 1:**
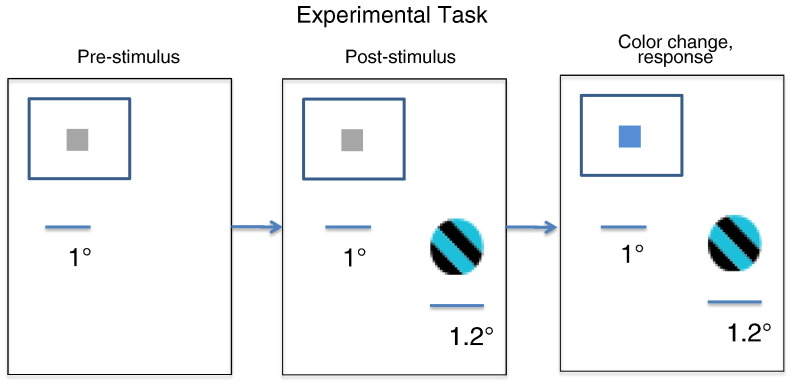
Experimental task. The monkey released a lever when it detected a colour change at fixation. The colour change could occur at any moment in time following fixation onset, starting from 200 ms post-fixation onset to 3 s post-fixation onset. Whilst the monkey fixated, a stimulus located at 4° of eccentricity was displayed to the monkey (a physically isoluminant sinusoidal grating; diameter: 1.2°; spatial frequency: 0.4-0.8 cycles/°; drift velocity: 0.6°/s drifting within a circular aperture). Stimulus contrast varied between zero and an 82% contrast value.

All experimental procedures reported in this study were approved by the ethics committee of the Radboud University, Nijmegen, NL. Details of the task and the surgical procedures have been described elsewhere (Bosman et al., 2012; Rubehn et al., 2009). In brief, stimuli were presented on a CRT (cathode ray tube) screen refreshing at 120 Hz non-interlaced and positioned such that 32 pixels corresponded to 1 degree of visual angle (°). Stimulus presentation, fixation and reward delivery were controlled by the *cortex* system. One adult male Rhesus monkey was trained to bring its gaze onto a fixation point at the centre of a computer monitor, and to keep its gaze within a window of 1° radius around the fixation point (see Fig. 1). The monkey's task was to release a lever when it detected a colour change at fixation. The colour change could occur at any moment in time from 200 ms post-fixation onset until 3 s post-fixation onset. Whilst the monkey fixated, a stimulus located at 4° of eccentricity was displayed (a physically isoluminant sinusoidal grating; diameter: 1.2°; spatial frequency: 1.7 cycles/s; drift velocity: 0.4°/s drifting within a circular aperture). Stimulus contrast was varied over the following values: 0%, 5%, 10%, 16%, 23%, 32%, 44%, 60% and 82%. These contrast levels were chosen such that from the 5% contrast condition onwards, each contrast condition approximately doubled the light intensity. The stimulus activated several contacts in V1. We chose contacts that showed strong gamma band activity (Bosman et al., 2012). The data from 200 ms after stimulus onset until fixation colour change were divided into non-overlapping 500 ms epochs for further analysis. The stimuli used here were similar to those of Chavane et al. (2011), who found that stimulus contrast does not affect the orientation selective spread. The resolution of our ECoG grid does not allow us to discriminate between different orientation preference columns: drifts in orientation are not likely to affect our results, as the responses we model are thought to result from an average across cortical patches with different orientation sensitivities. In other words, the population averaged activity measured by electrocorticography reports the average over orientation selective responses. This speaks to the use of neural field models as mathematical microscopes that allow one to extract information that is hidden in the data using DCM. Although our data are insensitive to orientation selective responses, we will see below that the use of a biophysically informed model allows us to infer that contrast dependent modulation of the effective spatial extent of neuronal connections contributes to the observed gamma response (a result that accords with single cell studies).

### Neurophysiological recordings, data analysis and ROIs

Neuronal signals were recorded from the monkey's left hemisphere using subdural electrocorticographic (ECoG) grids comprising 252 electrodes (1 mm diameter) spaced 2–3 mm apart (Rubehn et al., 2009). The grid was implanted under aseptic conditions with isoflurane anaesthesia supplemented with fentanyl. Intra-operative photographs were acquired for coregistration with the anatomy. Signals were amplified, low-pass filtered at 8 kHz and digitised at 32 kHz. Local field potentials were obtained by low-pass filtering at 250 Hz and down sampling to 1 kHz.

We computed bipolar differences from neighbouring electrodes to remove the common recording reference (Bosman et al., 2012). We refer to the bipolar channels as ‘sites’. We chose those sites that showed clear responses to the stimulus (a total of 4 sites), excluding immediate neighbours that shared a common unipolar electrode.

As noted above, we used data – in correctly executed trials – from 200 ms after the onset of the moving grating until the first change in the fixation point colour. This period constitutes relatively stationary visually induced activity. For each trial, this period was cut into non-overlapping 500 ms epochs (500 ms provides about five cycles of alpha activity, enabling reasonably efficient spectral estimators of alpha power). Data within a recording session were normalised for each site and subsequently pooled across all sessions. Power line artefacts at 50, 100 and 150 Hz were estimated and subtracted from the data. Epochs containing artefacts were removed with a semi-automatic artefact rejection protocol, based on a variance threshold. Sites were assigned to V1 based on their positions as ascertained from surgical photographs, and using established anatomical criteria; namely, that they were posterior to the lunate sulcus by at least 2 mm. We used the areal boundaries according to Saleem and Logothetis (2012). For the analyses of this paper, we use those sites in the primary visual cortex that showed high sensitivity to the stimuli.

#### Dynamic causal models for cross spectral densities

In this section, we describe our generative model of observed cross spectral densities that forms the basis of a dynamic causal model (DCM) with neural fields, used to model the above responses. This model is based upon canonical cortical microcircuitry and is a refinement of conventional (convolution-based) neuronal models. The ensuing field model is identical to established neural mass models in DCM that describe the sources of forward and backward connections in cortical hierarchies — the superficial and deep pyramidal cell populations respectively; however, it is equipped with horizontal connections that have an explicit spatial extent.

### A probabilistic model of cortical responses

The modelling of electrophysiological signals depends upon models of how they are generated in source space and how the resulting (hidden) neuronal states are detected by sensors. Following Pinotsis et al. (2012), we use a likelihood model relating hidden neuronal states to observed cross spectra *g_y_* over sensors that sample from the cortical surface. This likelihood model assumes the measured signal is a mixture of predicted spectra, channel and Gaussian observation noise

(1) gyω=g⌢ωθ+gnωθ+εyg⌢lmωθ=∑kTlkωgukωTmkω*guωθ=αu+βuωgnωθ=αn+βnωReε~N0,ΣωλImε~N0,Σωλ

where the indices *l* and *m* denote different sensors and * denotes the conjugate transpose matrix. The first equality expresses the data features *g_y_*(*ω*) as a mixture of predictions and prediction errors *ε_y_* with covariance Σ(ω,*λ*). The predictions are a mixture of predicted cross spectra $\overset{⌢}{g}\left(\omega ,\mu \right)$ and channel noise *g_n_*(*ω*,*μ*). The predicted cross spectra between two sites are a function of the power of underlying neuronal fluctuations *g_u_*(*ω*,*θ*) and transfer functions *T_l_*(*k*,*ω*) that depend upon model parameters *θ* encoding the neuronal architecture mediating responses (see below). The spectra of the neuronal fluctuations or inputs are modelled as a mixture of white and coloured components.

Eq. (1) provides the basis for our generative model and entails free parameters controlling the spectra of the inputs and channel noise {*α_n_*,*α_u_*,*β_n_*,*β_u_*} ⊂ *θ*. Gaussian assumptions about the observation error mean that we have a probabilistic mapping from the unknown parameters to observed (spectral) data features. Inversion of this model means estimating, probabilistically, the free parameters from the data.

At the neuronal level, we consider a neural field model based on the canonical microcircuit. This model differs slightly from the model described previously (Pinotsis et al., 2012) in that the pyramidal cell population is split into superficial and deep subpopulations. This separates the sources of forward and backward connections in cortical hierarchies and has proved useful when trying to explain several aspects of distributed cortical computations in theoretical neurobiology (Bastos et al., 2012; Buffalo et al., 2011). This model provides the particular form of the predicted spectra. In other words, the predicted cross spectral densities are specified by the transfer function associated with the neural field model and the mapping from source to sensor space, called the lead field. The transfer function depends on spatial and synaptic parameters that determine the spectral properties of observed activity (like peak frequency and power). We now consider the predicted responses as functions of the model parameters:

The predicted time series at the *l*-th ECoG sensor is given by

(2) $$y_{l}\left(t,\theta \right)=\int L_{l}\left(x,\varphi \right)\left(Q\cdot V\left(x,t\right)\right)\mathrm{dx}$$

Here, the lead field *L_l_*(*x*,*φ*) is a continuous function over the cortical patch (depending upon some parameters *φ* ⊂ *θ*) parameterising the sensitivity of the sensor to source activity. *V*(*x*,*t*)is a (vector-valued) function describing source activity in terms of the depolarisation or firing rate of several populations or cortical layers and *Q* = [*q*_1_*q*_2_*q*_3_*q*_4_] is a vector of coefficients that weights the relative contributions of these neuronal populations to the observed signal. This equation allows one to integrate out the dependence on the particular location on a Euclidean manifold or patch and obtain a time series at a single point — by summing up spatiotemporal activity over different spatial scales. For steady-state or evoked responses, this activity corresponds to plane waves of different wavelengths (parameterised by a wavenumber *k*). Expanding the lead field *L_l_*(*x*,*φ*) in terms of the coefficients *L_l_*(*k*,*φ*) of a spatial Fourier basis set, we have

(3) $$L_{l}\left(x,\varphi \right)=\sum_{k}L_{l}\left(k,\varphi \right)e^{\mathrm{ikx}}$$

Substituting this equality into Eq. (2), we obtain the temporal response at the *l*-th sensor

(4) $$\begin{array}{c} Y_{l}\left(\omega ,\theta \right)=\sum_{k}L_{l}\left(k,\varphi \right)\left(Q\cdot V\left(k,\omega \right)\right)\\ V\left(k,\omega \right)=T\left(k,\omega \right)U\left(k,\omega \right) \end{array}$$

Here, *U*(*k*,*ω*) is a spatiotemporal representation of fluctuations or inputs driving induced responses, which we assume to be spatially white. The response of the neuronal populations to this input is determined by the transfer function *T*(*k*,*ω*). Because we calculated local bipolar differences between LFPs from neighbouring electrodes to create sites, we modelled the lead field associated with each site using a difference of Gaussians — whose centres on the patch are given by the parameters *a_l_* and *a_j_*. These bipolar derivations are a special case of a generic montage operator *M* transforming responses from sensors to sites as follows

(5) $$\begin{array}{cc} \left[\begin{array}{c} y_{12}\\ y_{23}\\ \vdots \end{array}\right]=M\cdot \left[\begin{array}{c} Y_{1}\\ Y_{2}\\ Y_{3}\\ \vdots \end{array}\right], & M=\left[\begin{array}{cccc} -1 & 1 & 0 & \cdots \\ 0 & -1 & 1 & \cdots \\ 0 & 0 & -1 & \cdots \\ \vdots & \vdots & \vdots & \ddots \end{array}\right] \end{array}$$

Here, we use *y* to distinguish the time series associated with a site from the corresponding series *Y* detected by a sensor. Assuming that the cross spectrum of the inputs is given by *g_u_*(*k*,*ω*) = *U*(*k*,*ω*)*U*(*k*,*ω*)^⁎^, the predicted cross spectral density measured at sites *l* and *m* is given by

(6) $$\begin{array}{c} {\overset{⌢}{g}}_{\mathrm{lm}}\left(\omega ,\theta \right)=y_{l}\left(\omega ,\theta \right)y_{m}^{*}\left(\omega ,\theta \right)\\ =\sum_{k,q,p}T_{\mathrm{lq}}\left(k,\omega \right)g_{u}\left(k,\omega \right)T_{\mathrm{pm}}{\left(k,\omega \right)}^{*}\\ T_{\mathrm{lq}}\left(k,\omega \right)=M_{\mathrm{lq}}L_{q}\left(k,\varphi \right)Q\cdot T\left(k,\omega \right) \end{array}$$

where *L_q_*(*k*,*φ*) is the Fourier transform of the lead field of the sensor *q*. In this setting, each electrode or sensor sees only part of the patch, with its sensitivity reflecting its relative position with respect to the highest source activity.

For each contrast condition, we analysed data from four sites at varying distances from the focus of induced activity. We equipped our observation model with sufficient degrees of freedom to model the sensitivity of each site. The important thing here is that to explain differences in observed responses, we need to concurrently optimise neuronal parameters and parameters encoding the (unknown) deployment and sensitivity of sensors on the cortical surface. These parameters include the centre of each source and the dispersion of its lead field (the actual sensor positions on the cortex were of course fixed but the location of the underlying neural field is not known).

The model predictions are given by Eq. (6), which holds for a general montage operator. For the bipolar difference form in Eq. (5), the cross spectral densities amongst measurement sites take the following form

(7) $$\begin{array}{c} {\overset{⌢}{g}}_{\mathrm{lm}}\left(\omega ,\theta \right)=\sum_{k}{\tilde{T}}_{l}\left(k,\omega \right)g_{u}\left(k,\omega \right){\tilde{T}}_{m}{\left(k,\omega \right)}^{*}\\ {\tilde{T}}_{l}\left(k,\omega \right)=\left(e^{-ia_{l}k}E\left(\phi_{l}\right)-e^{-ia_{l-1}k}E\left(\phi_{l-1}\right)\right)Q\cdot T\left(k,\omega \right)\\ E\left(\phi \right)=e^{-2\pi^{2}\phi^{2}k^{2}} \end{array}$$

where *a_i_*,*a*_*i* − 1_,⊂*θ* are the centres of the two electrodes associated with the *i*-th site and *ϕ_i_*,*ϕ*_*i* − 1_,⊂*θ* are the corresponding dispersions. These parameters are optimised depending on the location of the corresponding electrode and sensitivity profile.

The likelihood model defined by Eqs. (1) and (7) can furnish predictions for conventional measures of linear systems; like coherence, phase delay or cross correlation functions, as detailed in Friston et al. (2012). One can then examine the influence of biophysical model parameters like synaptic time constants and intrinsic conduction speed on these classical measures. One can also exploit these characterisations to inform and constrain biophysical parameters, like conduction delays. In brief, there is a mapping between model parameters (effective connectivity) and spectral characterisations (functional connectivity) that provides a useful link between the generative modelling of biophysical time series and dynamical systems theory. One should however be careful in interpreting estimates of phase delays as an expression of conduction delays: although it is tempting to assume a direct correspondence between these two measures, their relationship is complicated because phase delays can differ between frequencies, whilst conduction delays are determined by axonal microarchitecture and do not change in frequency (Friston et al., 2012).

The above discussion completes the description of the generative model apart from the transfer functions. The transfer function *T*(*k*,*ω*) depends on cortical microcircuitry, which is derived below by appealing to canonical microcircuitry. We briefly review the properties of this model and then turn to its mathematical formulation.

### Spectral asymmetries and canonical cortical microcircuitry

Microelectrode recordings of spikes and local field potentials in visual areas V1, V2 and V4 suggest that neurons in superficial layers synchronise at gamma frequencies, whilst neurons in deep layers primarily synchronise at alpha/beta frequencies (Buffalo et al., 2011). Since forward connections originate predominately from superficial layers and backward connections primarily originate in deep layers (Felleman and van Essen, 1991), these spectral asymmetries suggest that forward connections use higher (gamma) temporal frequencies, whilst backward connections may employ lower (alpha or beta) frequencies. Indeed, simultaneous recordings in monkey areas V1 and V4 have demonstrated that Granger causal influences in the gamma-frequency band are primarily feedforward (Bosman et al., 2012). In vitro recordings provide some support for a predominance of beta-band influences in the feedback direction (Roopun et al., 2010). These asymmetries suggest something quite remarkable: each macrocolumn receives forward input at gamma and backward input at beta. It then integrates both and emits forward output at gamma and backward output at beta. In other words, it integrates and segregates its inputs and outputs using two distinct frequency channels. These cortical computations face (at least) four challenges: First, afferent input to a macrocolumn is typically weak compared to intrinsic activity. For example, in cortical area V1, only 4% of all the synapses in the granular (main input) layer are from the LGN — the remaining synapses are local intrinsic connections (Binzegger et al., 2004). The cortex needs mechanisms to effectively select and sustain these sparse inputs. Second, as the cortex amplifies these inputs, strict homeostatic circuit properties must be in place to constrain excitation – relative to inhibition – to prevent runaway excitation: as observed experimentally by Haider et al. (2006). Third, a given cell in the cortical column must be able to effectively select relevant synaptic inputs from a massive number of potentially irrelevant signals, since a given pyramidal cell in the cortex receives about 10,000 synapses (Larkman, 2004). Fourth, in order to functionally segregate top-down from bottom-up processing, a given column must be able to separate higher-order inputs from lower-order inputs — although this appears to be finessed by the laminar termination of synaptic inputs (Felleman and Van Essen, 1991). Inputs to a cortical column from cortical areas above it in the hierarchy could, through their larger sampling of the perceptual field and their more elaborated response properties, convey messages that contextualise signals arising from earlier areas. These computational challenges are faced by nearly all cortical areas. If a solution to these issues arose during evolution, it seems likely that it would be conserved and present, to some extent, in all cortical circuits.

The canonical microcircuit model first proposed by Douglas and Martin (2004) contains all the requisite properties to satisfy these computational demands. In their model, weak thalamic inputs project onto a cortical column containing three cell populations: excitatory cells in the superficial and deep cortical layers, and a common pool of inhibitory interneurons. Through intrinsic interconnections amongst these populations, weak thalamic inputs are amplified. Reciprocal connections between the populations maintain a balance of inhibition and excitation. Relatively strong connections between the inhibitory cells and deep pyramidal cells segregate the superficial and deep cell responses, in terms of their latency. Lastly, in their revised model, dense (lateral) interconnections amongst the superficial pyramidal cells allow these cells to sample their diverse inputs on the dendritic tree and implement a version of a winner-take-all algorithm (Douglas and Martin, 2004). The results of this computation are transferred to lower cortical areas via the deep pyramidal cells or higher cortical areas via the superficial cells.

Models for canonical cortical circuitry have become increasingly complex as researchers have characterised cortical circuits using anatomical and functional methods to elucidate the precise patterns of intrinsic connections. For example Thomson and Bannister (2003) combined multiple whole-cell recordings with histology to identify the probability of finding interconnected cells between particular layers, and their synaptic strengths. Their work underlines the importance of a ‘feedforward’ pathway for how information spreads in the cortex through excitatory connections. Inputs enters mostly layer 4, where a strong connection is sent to superficial cells in Layer 3 (L3). L3 is strongly interconnected with other pyramidal cells. The main output of L3 is onto the infragranular cells in L5. Thus, input to superficial, and superficial to deep, along with strong intra-laminar connections, appear to be canonical features of intrinsic connectivity. Whilst this core circuit has been established mostly for primary visual cortex (which is also our focus here), recent studies have also demonstrated that other cortical areas such as primary somatosensory and primary motor cortex share similar circuit properties (Lefort et al., 2009; Weiler et al., 2008).

**Fig. 2:**
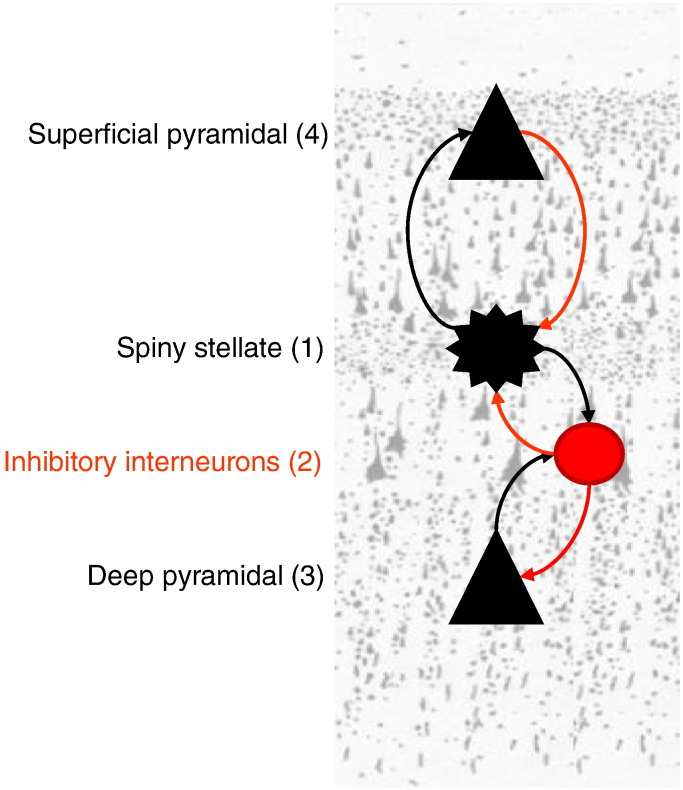
The canonical microcircuit (CMC) model. This model comprises four subpopulations and its development was motivated by theoretical considerations about hierarchical message passing and asymmetries of oscillation frequencies in the brain and its architecture is based upon intracellular recordings in cat visual cortex. This canonical microcircuit incorporates the neuronal sources of forward and backward connections in cortical hierarchies. These are the distinct superficial and deep pyramidal cell populations where superficial populations generate gamma responses whilst deep populations generate slower (alpha and beta) dynamics. The colours of the arrows correspond to excitatory (black) and inhibitory (red) connections respectively. The numbers in parentheses next to each cell-name serve as indices of the corresponding populations and their connections.

Haeusler and Maass used Hodgkin and Huxley neurons to build a realistic microcircuit model and showed that a cortical column – whose connectivity conforms to the canonical microcircuit – can perform various computations more efficiently, in relation to a column with random connectivity (Haeusler and Maass, 2007). Bastos et al. (2012) compared the canonical microcircuit model from the Haeusler and Maass model to the theoretically-predicted circuit based on predictive coding (Friston, 2008). By collapsing two pairs of populations in the Bastos et al. (2012) model – whilst preserving the topology of the connectivity – one obtains the circuit of Fig. 2, which comprises four populations: excitatory spiny stellate input cells (1), inhibitory interneurons (2), deep excitatory output pyramidal cells (3) and superficial excitatory pyramidal cells (4). The arrangement in Fig. 2 can be regarded as a formal proposal for a canonical microcircuit. In what follows, we describe a mathematical model of how the neuronal states of these populations evolve over time on the cortical surface.

### A canonical field model of cortical activity

In neural field models, populations comprising a cortical source are assumed to occupy Euclidean manifolds (infinitely thin planes) that are coupled by interlaminar or intrinsic connections. The canonical microcircuitry described above prescribes connectivity rules between planes or subpopulations comprising a macrocolumn that can be described in terms of a connectivity matrix *K*(|*x*|)

(8) $$K\left(\left|x\right|\right)=\frac{1}{2}\left[\begin{array}{cccc} -\alpha_{11}e^{-c_{11}|x|} & -\alpha_{12}e^{-c_{12}|x|} & 0 & -\alpha_{14}e^{-c_{14}|x|}\\ \alpha_{21}e^{-c_{21}|x|} & -\alpha_{22}e^{-c_{22}|x|} & \alpha_{23}e^{-c_{23}|x|} & 0\\ 0 & -\alpha_{32}e^{-c_{32}|x|} & -\alpha_{33}e^{-c_{33}|x|} & 0\\ \alpha_{41}e^{-c_{41}|x|} & 0 & 0 & -\alpha_{44}e^{-c_{44}|x|} \end{array}\right]$$

In this expression, *α_ab_* > 0 are synaptic coupling strengths that describe the density of intrinsic connections and the population response to presynaptic glutamate release by pyramidal and spiny stellate cells and GABA by interneurons. Here, *c_ab_* > 0 encode the spatial decay of synaptic densities — assuming an exponential form. This is a ubiquitous choice in the neural field literature, motivated by the sparse (heavy tailed) distribution of horizontal connections in sensory cortices: for more details, see (Baker and Cowan, 2009). Later, we will use the parameters *c_ab_* to describe the spatial extent of the receptive fields of excitatory and inhibitory populations. Assuming that *v_a_*(*x*,*t*) denotes the expected depolarisation or firing rate of the *a*-th population or layer (*a* = 1,…,4) at location *x* and time *t*, the time evolution of the vector *V* = (*v*_1_,*v*_2_,*v*_3_,*v*_4_,)*^T^* – describing their depolarisation – has the following form:

(9) $$\begin{array}{c} \left(\ddot{V}+2B\dot{V}+B^{2}V\right)(x,t)=B\int K\left(x-x^{\prime }\right)F\circ V\left(x^{\prime },t-\left|x-x^{\prime }\right|\upsilon \right)dx^{\prime }+G\circ U\\ B=\mathrm{diag}\left(\kappa_{1},\kappa_{2},\kappa_{3},\kappa_{4}\right) \end{array}$$

where *υ* is the inverse speed with which spikes propagate along connections, *U*(*x*,*t*) is input (neuronal fluctuations) and *κ*_*a*_ are (inverse) synaptic time constants mediating postsynaptic filtering. In other words, the response of each population results from passive membrane properties and dendrite dynamics. Also, *G* : *R*^4^ → *R*^4^ maps the inputs to the motion of hidden neuronal states, *G* = (*κ*_1_, 0, 0, 0)^*T*^ and *F* : *R*^4^ → *R*^4^ is a nonlinear mapping from postsynaptic depolarisation to presynaptic firing rates, which we take to be a sigmoid

(10) Fvi=11+exprη−vi

Here, *r* and *η* are parameters that determine the shape of this sigmoid. In particular, *r* is the synaptic gain and *η* is the postsynaptic potential that produces half of the maximum firing rate. Following Pinotsis et al. (2012), we obtain the transfer function associated with the above system of neural field equations

(11) $$T\left(k,\omega \right)={\left(-\omega^{2}I_{4}-2\mathrm{i\omega B}+B^{2}-J\left(k,\omega \right)\right)}^{-1}G$$

where *I*_4_ is the identity matrix and *J*(*k*,*ω*) is a 4 × 4 matrix incorporating spatial and synaptic parameters, connectivity densities and synaptic gain matrix:

(12) $$\begin{array}{c} J\left(k,\omega \right)=\mathrm{BD}\left(k,\omega \right)\gamma \\ \gamma_{\mathrm{ab}}=\frac{\partial F\left(v_{a}=0\right)}{\partial v_{b}}=\left\{\begin{array}{cc} \frac{re^{\mathrm{r\eta}}}{{\left(1+e^{\mathrm{r\eta}}\right)}^{2}} & a=b\\ 0 & a\neq b \end{array}\right)\\ D_{\mathrm{ab}}\left(k,\omega \right)=\frac{a_{\mathrm{ab}}\left(c_{\mathrm{ab}}-\mathrm{i\upsilon \omega}\right)}{c_{\mathrm{ab}}^{2}-\upsilon_{\mathrm{ab}}^{2}\omega^{2}-2\mathrm{i\upsilon}c_{\mathrm{ab}}\omega +k^{2}} \end{array}$$

Substituting Eqs. (11) and (12) in Eq. (7), we obtain the following expression for the transfer function of each population

(13) $$T_{a}\left(k,\omega \right)=\kappa_{1}W^{-1}\left(k,\omega \right)Z_{a}\left(k,\omega \right)$$

This expresses the relative contribution of each population to the predictions at the sensor level and depends upon the particular form of the connections amongst source populations, where *W*(*k*,*ω*) and *Z_a_*(*k*,*ω*) are given by

(14) $$\begin{array}{c} W\left(k,\omega \right)=-R_{14}\left(k,\omega \right)\left(-R_{23}\left(k,\omega \right)+P_{3}\left(k,\omega \right)P_{2}\left(k,\omega \right)\right)\\ +P_{4}\left(k,\omega \right)\left[-R_{23}\left(k,\omega \right)P_{1}\left(k,\omega \right)+P_{3}\left(k,\omega \right)\left(-R_{12}\left(k,\omega \right)+P_{2}\left(k,\omega \right)P_{1}\left(k,\omega \right)\right)\right]\\ Z_{1}\left(k,\omega \right)=-P_{4}\left(k,\omega \right)\left(-R_{23}\left(k,\omega \right)+P_{3}\left(k,\omega \right)P_{2}\left(k,\omega \right)\right)\\ Z_{2}\left(k,\omega \right)=D_{21}\left(k,\omega \right)\gamma \kappa_{2}P_{4}\left(k,\omega \right)P_{3}\left(k,\omega \right)\\ Z_{3}\left(k,\omega \right)=-D_{21}\left(k,\omega \right)D_{32}\left(k,\omega \right)\gamma^{2}\kappa_{2}\kappa_{3}P_{4}\left(k,\omega \right)\\ Z_{4}\left(k,\omega \right)=D_{41}\left(k,\omega \right)\gamma \kappa_{4}\left(-R_{23}\left(k,\omega \right)+P_{3}\left(k,\omega \right)P_{2}\left(k,\omega \right)\right) \end{array}$$

and the functions *P_a_*(*k*,*ω*) and *R_ab_*(*k*,*ω*) are given in terms of the Fourier transform *D_ab_*(*k*,*ω*) as follows:

(15) $$\begin{array}{c} P_{a}\left(k,\omega \right)=2i\kappa_{a}\omega +\omega^{2}-{\kappa_{a}}^{2}+\gamma D_{\mathrm{aa}}\left(k,\omega \right)\kappa_{a}\\ R_{\mathrm{ab}}\left(k,\omega \right)=\gamma^{2}\kappa_{a}\kappa_{b}D_{\mathrm{ab}}\left(k,\omega \right)D_{\mathrm{ba}}\left(k,\omega \right) \end{array}$$

This completes the mathematical specification of the likelihood model that maps from neuronal fluctuations driving cortical layers to cross spectral densities in an array of sensors. To complete the specification of the dynamic causal model we now need to consider the constraints or priors on its underlying parameters.

### The generative model and its inversion

Substituting the expression for predicted responses in the frequency domain in Eq. (7), into Eq. (1), furnishes a likelihood model for measured spectral responses. In the following, we use this model to analyse complex cross spectra in V1, as measured with ECoG arrays. We assume that the visual cortex is tiled with macrocolumns and that the response of each local source or patch can be described in terms of a receptive field with rotational symmetry. This receptive field depends upon the topography of the neuronal connections and its orientation axis coincides with the coordinates of the field model. Below, we use parameters encoding the range of inhibitory and excitatory source components to characterise spatial summation of receptive fields based on (mean field) gamma activity. Furthermore, we will examine estimates of neuronal parameters to characterise the excitatory and inhibitory postsynaptic potentials (EPSPs, IPSPs) elicited by horizontal connections under different levels of visual contrast. The underlying assumption here is that the cortex acts as a dynamic filter of visual stimuli — that shows rapid nonlinear adaptation and where the local centre–surround interactions determine the frequency of gamma oscillations (Kapadia et al., 1999). Technically speaking, we assume that spectral responses result from perturbations around a zero fixed point. This means that a change in the parameters only changes the system's flow (and implicitly the Jacobian and associated transfer functions) but not the expansion point. This is a valid assumption for the convolution based neural field model used in this paper; however, things get more complicated with conductance based models, where the expansion point itself can change with the parameters. We consider contrast-specific effects and simultaneously optimise responses obtained from all conditions across sites that show stimulus induced responses. As noted above, the exact locations of each site on the cortical patch are also optimised during model inversion or fitting of the cross spectral data. These data inform the spatial sensitivity of the recording sites by allowing for conduction delays and other spatial effects including the relative location of the sensors. We distinguish between parameters describing the extent of excitatory and inhibitory populations and assume a conduction velocity of 0.3 m/s (Nauhaus et al., 2008).

Clearly, there are a large number of parameters in these models. However, many of these parameters are subject to physiologically plausible constraints — such that they do not over fit the data. Parameter estimation and evaluation of the evidence for competing models rests upon Bayesian model inversion that calls for a formal specification of prior constraints of the parameters. Table 1 describes the priors over synaptic parameters (as used in the classical Jansen and Rit model), as well as parameters pertaining to the spatial organisation of cortical sources. These priors are based on the modelling literature, whilst others come from the experimental literature. The priors of the lead field model assume the sensors are located close to the middle of the patch and at distance *ℓ*/8 apart. During model optimisation, these prior means are multiplied by scale factors with lognormal priors.

The likelihood *p*(*g_y_*|*θ*,*m*) in Eq. (1) and the priors *p*(*θ*|*m*) specify a dynamic causal model that can be inverted using standard variational procedures (Friston et al., 2007). For any DCM, say model *m*, model inversion appeals to Bayes rule

(16) $$p\left(\theta |g_{y},m\right)=\frac{p\left(g_{y}|\theta ,m\right)p\left(\theta |m\right)}{p\left(g_{y}|m\right)}$$

where *p*(*g_y_*|*m*)is the model evidence. Model inversion rests upon a fixed-form Laplace assumption $q\left(\theta \right)=N\left(\mu ,C\right)$ for the posterior density^2^ over unknown parameters. This Variational Laplace scheme approximates model evidence with a variational free energy. The (approximate) posterior density and (approximate) log evidence are used for inference on parameters and models respectively. In other words, one can compare different models (e.g., neural field and mass models) using their log evidence and also make inferences on parameters, under the model selected. A full description of these schemes can be found in Friston et al. (2007).

## Results

In what follows, we report the results of Bayesian model comparison — using the variational free energy approximation to model evidence. First, we assess the evidence for spatially organised source activity by comparing neuronal mass and field models. We then proceed to address our questions about the nature of cortical gain control by examining different models of contrast dependent changes in intrinsic and horizontal connectivity.

**Fig. 3:**
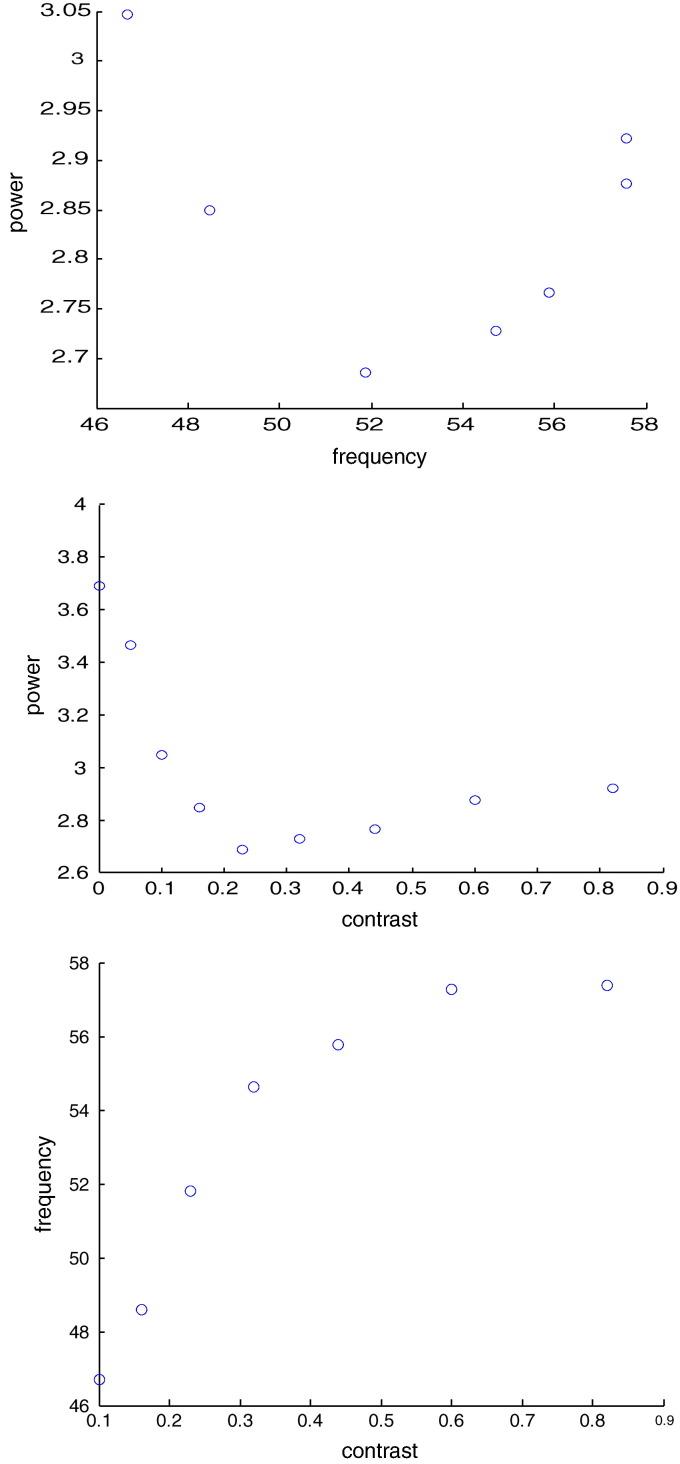
Data features of interest. The middle subpanel shows the gamma peak frequency as a function of the different contrast levels which were presented. Note that a gamma peak was not detected in the 0% contrast condition (no visual stimulation to the receptive field) nor reliably in the 0.05% contrast condition, and these are therefore omitted. The upper panel shows a U relationship between gamma peak frequency and power in the gamma band (30–80 Hz). The middle subpanel shows the relationship between contrast and gamma band power, for all nine experimental conditions.

**Fig. 4:**
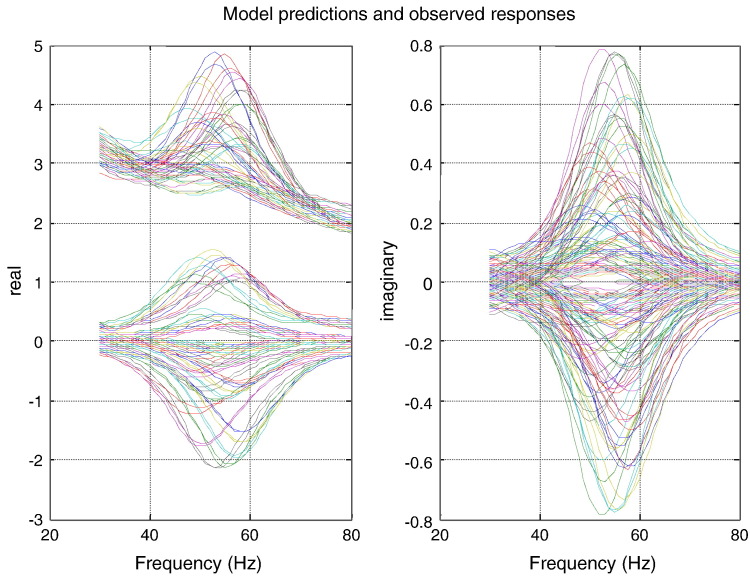
Model predictions and data for all conditions simultaneously. Empirical responses and model fits are shown in dashed and solid lines. The real and imaginary parts of model fits and observed spectral responses are shown in the left and right panels respectively. In the left panel, the two sets of curves correspond to auto- and cross spectral densities. Subsequent model comparisons rely on model evidence in data from all conditions.

Before turning to model optimisation, we first characterised some of the key data features we were hoping to explain. Specifically, we quantified the relationship between visual contrast, gamma power and the peak frequency of induced responses: we found a U relationship between observed peak frequency and gamma power and between power and contrast (see Fig. 3). Crucially, peak frequency increases as a monotonic function of contrast (Fig. 3, top panel). Fig. 4 shows an example of a DCM fit to cross spectral responses obtained following model inversion. These show the real (left panel) and imaginary (right panel) spectra and cross spectra, from all conditions and over all pairs of sites. The agreement between the model predictions (full lines) and empirical spectra (dotted lines) is self-evident.

### Evidence for neural fields

**Fig. 5:**
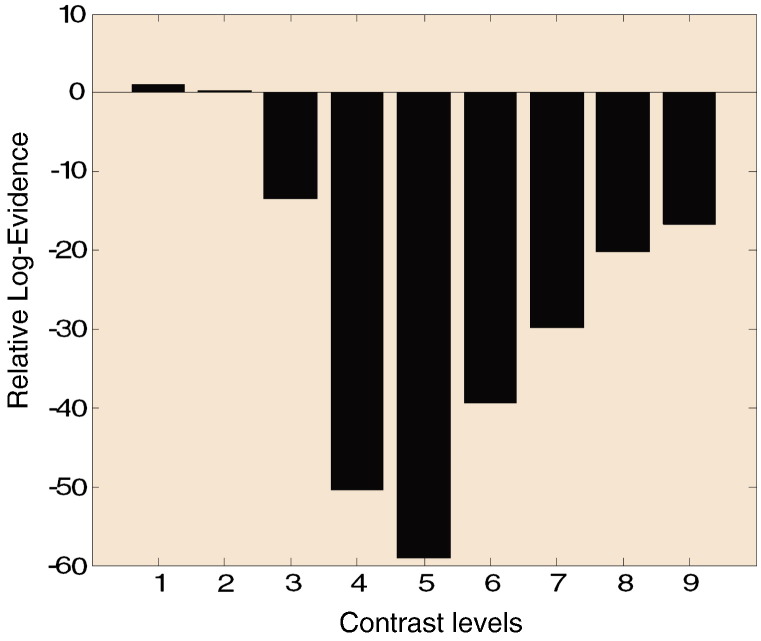
Relative log evidence for mass and field models. For the two conditions of lowest contrast there is no clear evidence in favour of one of the two models, whilst for the remaining conditions Bayesian model comparison favours neural fields.

The advantage of neural field models is that they can accommodate spatially extended activity on cortical manifold or patches that endows the predicted responses with a complicated frequency dependency (for a further discussion, see Pinotsis et al., 2012). This allows one to distinguish between spatial effects and other factors (such as intrinsic cell properties) on the basis of observed (empirical) responses. In brief, our Bayesian model comparison suggested that neuronal field models provide better explanations for ECoG data than the equivalent neural mass models:

We computed the relative log evidence for the neural field and mass variants of the canonical microcircuitry depicted in Fig. 2. The neural mass variant is identical to the neural field model but uses fixed priors on axonal conduction velocity that effectively shrink the cortical patch to a point. Fig. 5, presents the relative log evidence for neural mass and field models for various contrast conditions. We found very strong evidence in favour of the field model across high contrast conditions: a log evidence difference of three or more can be taken as very strong evidence and corresponds to a likelihood ratio of about 20:1. Our data suggest that there was no induced gamma peak for the two lowest contrasts and the corresponding spectra were rather featureless: but see also (Maris et al., 2013). In these two conditions, there was no clear evidence in favour of one of the two alternative models — with and without spatial dynamics. However, the remaining conditions were characterised by a distinct frequency peak and Bayesian model comparison strongly supported spatiotemporal (field) dynamics.

This result is in stark contrast with the analysis of (Pinotsis et al., 2013a). In this earlier work, we used MEG data from 16 human subjects exhibiting visually induced gamma oscillations. These data failed to establish a greater evidence for neural field models, which can be attributed to the lead fields inherent in non-invasive electromagnetic recordings — that are necessarily broader and therefore suppress temporal dynamics that are expressed in high spatial frequencies. In contrast, the spatiotemporally resolved information afforded by ECoG data (combining the temporal sensitivity of EEG with wide brain coverage and high spatial resolution) discloses a broad spectrum of spatiotemporal dynamics and provides strong evidence for induced activity over neural fields.

### Bayesian model selection

Model comparison uses the evidence for models in data from all conditions simultaneously. The models we compared allowed only a subset of parameters to vary with contrast level, where each model corresponds to a hypothesis about contrast-specific effects on cortical responses. Specifically, (the log of) contrast dependent parameters were allowed to change linearly with the contrast level, such that the effect of contrast was parameterised by the sensitivity of contrast dependent parameters to contrast level. The first set of models allowed contrast dependent gain of pyramidal cells (*a*_33_ and *a*_44_). Previous dynamic causal modelling has shown contrast dependent increases in the gain of superficial pyramidal cells in cortical hierarchies (Brown and Friston, 2012). This finding fits comfortably with recent neurobiological theories of attention and the hypothesis that contrast manipulation leads to changes in the precision of sensory inputs. These changes have been interpreted – in the context of predictive coding – as changes in the gain of superficial pyramidal cells reporting prediction error (Feldman and Friston, 2010).

The second set of models allowed for contrast dependent effects on the strengths of intrinsic connections between pyramidal cells, interneurons and spiny stellate cells (*a*_14_, *a*_12_, *a*_21_, *a*_23_, *a*_41_ and *a*_32_). This speaks to variations in the balance of cortical excitation and inhibition that is mediated by the relative sensitivity of different populations to interlaminar exchanges. The final set of models allowed for changes in the spatial extent of intrinsic (horizontal) excitatory and inhibitory connections (*c_ab_*) — that corresponds to a change in the effective connectivity mediating spatial summation of the receptive field. These connections serve stimulus integration and can be differentially engaged depending on stimulus properties.

These three putative mechanisms of gain control speak to models with and without contrast dependent effects on: (i) recurrent connections of neuronal populations, (ii) horizontal connections between excitatory and inhibitory pools of neurons and (iii) spatial dispersion of horizontal connections. These three model factors lead to 8 candidate models (or seven models excluding a model with no contrast dependent effects)

**Fig. 6:**
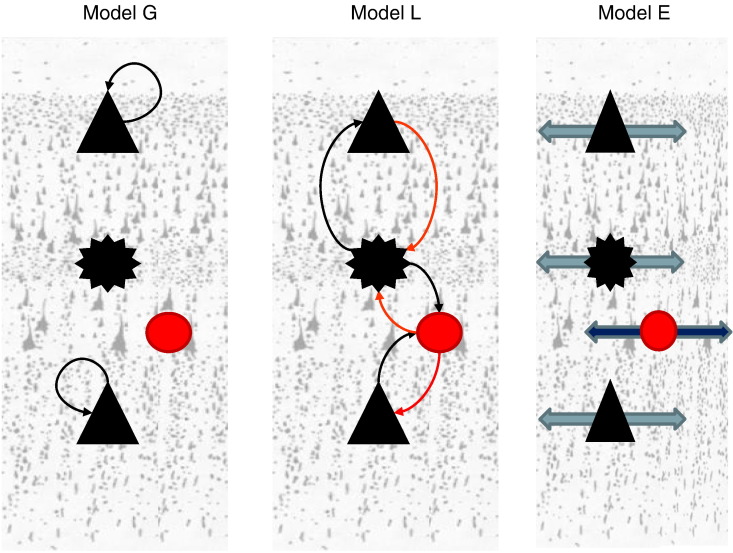
Candidate models. We consider three putative mechanisms of gain control that lead to models or hypotheses about trial-specific modulations of (G:) recurrent connections of neuronal populations, (L:) horizontal connections between distinct neuronal subpopulations and (E:) spatial dispersion of horizontal connections.

**Fig. 7:**
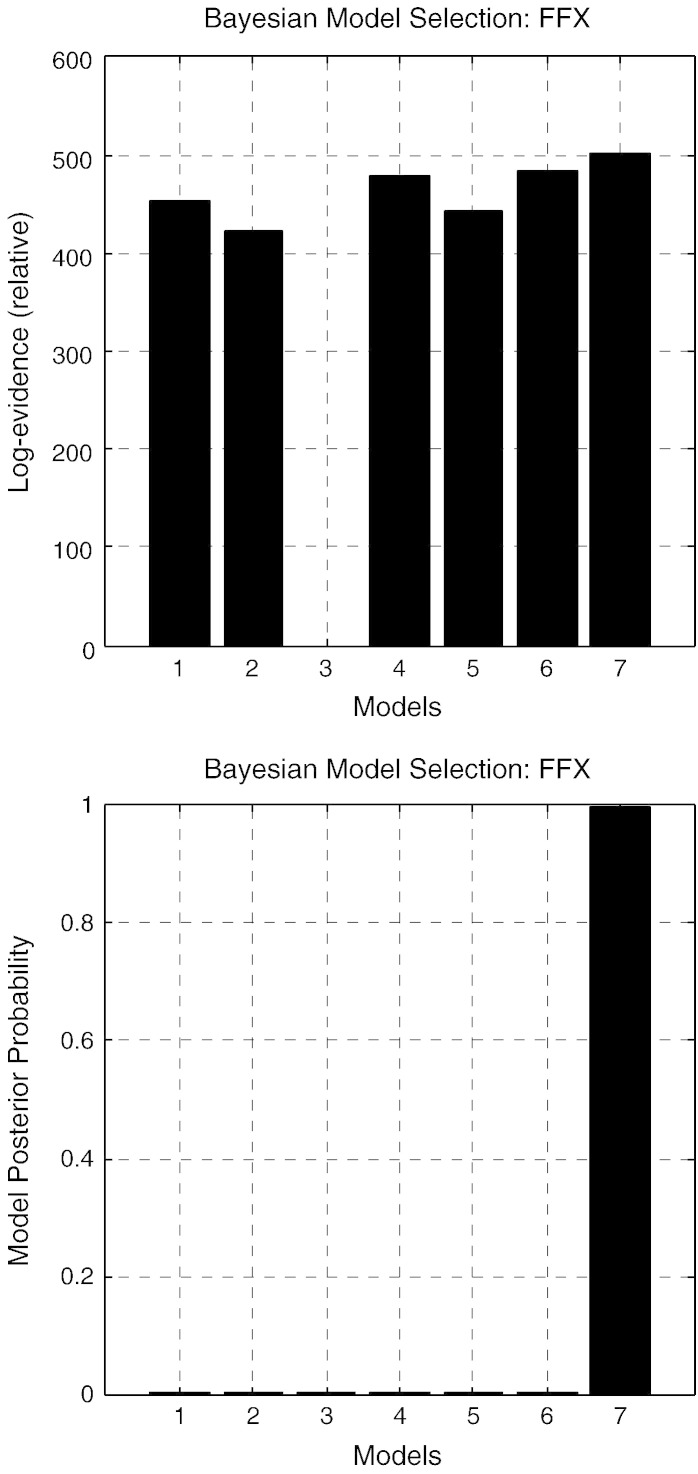
Bayesian model comparison. The model involving modulations of all parameters (model 7) has the highest evidence with a relative log evidence of 17 with respect to the model that allows for modulations in all but the extent parameters (model 6). The first three models correspond to hypotheses (i), (ii) and (iii) whilst models 4 and 5 to combinations (i) + (ii) and (ii) + (iii). In the bottom panel we include model posterior probabilities. These results suggest that we can be almost certain that all three synaptic mechanisms contribute to the formation of cross spectral features.

Fig. 6, shows three of the models that we considered. The seven (non-null) candidate models include the models depicted in Fig. 6 and their combinations. Our analysis amounts to a fixed effects analysis that rests on multiplying model evidences over conditions to give the total evidence for each model. The relative log evidences for all models are shown in Fig. 7: The model with contrast dependent effects on all parameters (model 7) had the highest evidence with a relative log evidence difference of 17 with respect to the model that allows for modulations of all but the extent parameters (model 6). In these plots, the first three models correspond to hypotheses (i), (ii) and (iii) whilst models 4 and 5 to combinations (i) and (iii) and (ii) and (iii). In the bottom panel, we see the corresponding model posterior probabilities (assuming uniform priors over all models considered). This suggests that we can be almost certain that all three synaptic mechanisms contribute to the formation of cross spectral density features observed under different levels of visual contrast (given the models evaluated).

**Fig. 8:**
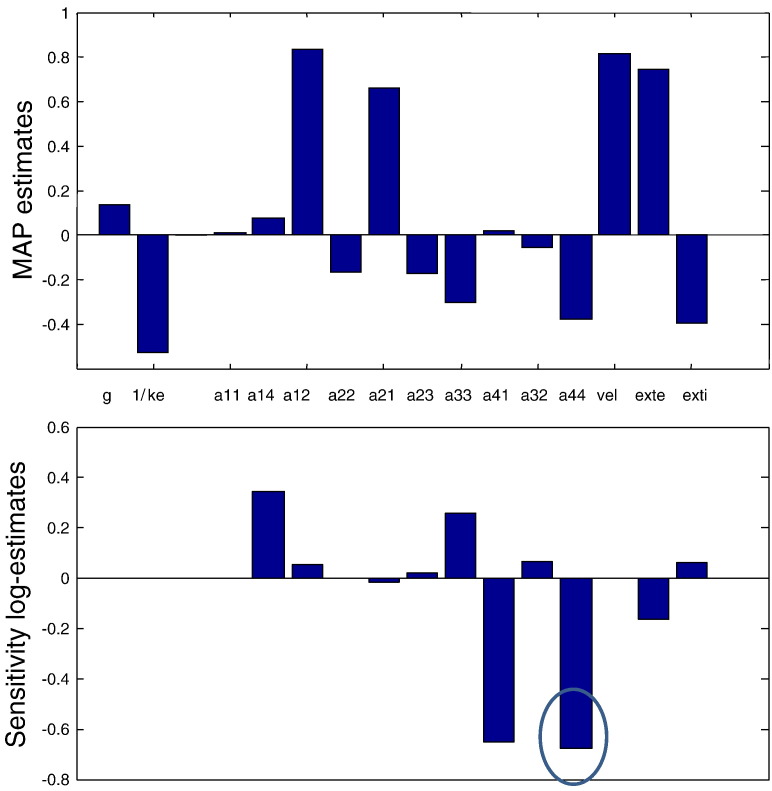
Parameter estimates and their changes: (top) Maximum a posteriori estimates of model parameters obtained after inverting model (vii). (Bottom) Corresponding sensitivity estimates of contrast dependent parameters. The largest contrast modulations are observed in estimates of connections to and from the superficial pyramidal cells (encircled).

Having established the best model, we then examined its parameter estimates: the maximum a posteriori estimates are shown in Fig. 8 (top), whilst estimates of their contrast sensitivity are shown in the lower panel. These results suggest that the largest contrast modulations are observed in (log scale parameter) estimates of connections to and from the superficial pyramidal cells (*a*_14_, *a*_41_ and *a*_44_). In particular, the largest variation over contrast is observed for the parameter *a*_44_ that corresponds to the gain associated with superficial pyramidal cells. Here, an increase in contrast reduces the inhibitory self connection leading to a disinhibitory increase in gain. This result is in accord with the predictive coding formulation above (see Brown and Friston, 2012), where the gain of superficial pyramidal cells is thought to encode the precision of prediction errors. As contrast increases, confidence in (precision of) sensory information rises. In predictive coding this is thought to be accompanied by an increase in the weighting of sensory prediction errors that are generally thought to be reported by superficial pyramidal cells.

The contrast dependent changes in the extent of horizontal connectivity suggest an effective shrinking of excitatory horizontal influences and an increase in inhibitory effects. This is precisely what would be expected on the basis of contrast dependent changes in receptive field size. As contrast increases, receptive field sizes shrink — effectively passing higher frequency information to higher levels. In other words, the neural field has a more compact spatial summation that depends upon gain control and the balance of horizontal excitatory and inhibitory connections.

**Fig. 9:**
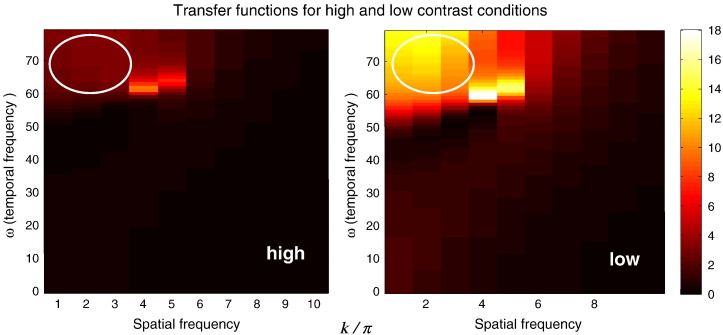
Transfer functions of superficial pyramidal cells for the highest (left) and lowest (right) contrast level. Temporal and spatial frequencies are shown in the vertical and horizontal axes (in Hz and in radians per unit distance respectively). These plots show that an increase in stimulus contrast leads to a decrease in the relative power of low spatial frequencies at gamma frequencies (in the upper left corner of the transfer function — encircled).

To quantify this contrast dependent change in spatial summation, we evaluated the spatiotemporal transfer function associated with the superficial pyramidal cell population *T*_4_(*k*,*ω*) for the parameter estimates shown in Fig. 8. We repeated this for the highest and lowest levels of contrast used and displayed the result as the absolute value squared over spatial and temporal frequencies (Fig. 9). This characterisation of the response properties of visual cortex regards the cortical layers as a spatiotemporal filter and allows one to quantify the spatial frequencies that are preferentially preserved as a function of the temporal frequencies.

One can see that low spatial frequencies are passed preferentially and – crucially – increasing the contrast decreases the relative amount of low spatial frequencies in the input at fast (gamma) frequencies. These transfer functions describe activity that is passed from one population to the other, within the local cortical circuit (not the observed response). In this instance, the inputs are the spiny stellate cell population and the (feedforward) output corresponds to the response of the superficial pyramidal cells. These results suggest that faster temporal (gamma band) frequencies observed empirically reflect the fact that superficial pyramidal cells pass less gamma power with increasing contrast (see Fig. 3) but are driven by relatively higher spatial frequencies: note the selective suppression of power at spatial frequencies less than 4 in the upper quadrant of the high contrast (left panel) transfer function. Note that one cannot divorce spatial and temporal filtering when neuronal infrastructure has spatial extent and this empirically informed spatiotemporal characterisation can only be quantified with a suitably formulated model of neuronal transformations — as provided by neural field models.

## Conclusion

In conclusion, we have used finely sampled electrophysiological responses from awake-behaving monkeys to quantify the microarchitecture of visual cortex, in terms of synaptic rate constants and intrinsic connectivity. Specifically, we have used an experimental manipulation (the contrast of visual stimuli) to look at changes in the gain and balance of excitatory and inhibitory influences. Our results suggest that increasing contrast effectively increases the sensitivity or gain of superficial pyramidal cells to inputs from spiny stellate populations. Furthermore, changes in the effective spatial extent or reach of horizontal coupling change the spatiotemporal filtering properties of cortical lamina in V1 to – effectively – preserve higher spatial frequencies.

Many readers will note that the particular model of canonical microcircuitry used in this paper is somewhat simpler than the canonical microcircuits considered by Haeusler and Maass — or reviewed from the perspective of predictive coding in Bastos (2013). These simplifications rest upon pooling various subpopulations to simplify the intrinsic connectivity — to ensure dynamical stability and more robust model inversion. The motivation for these simplifications – and the elaboration of the corresponding CMC model – will be considered in detail in a forthcoming publication (see also Bastos: Ph.D. thesis). The extent to which these simplifications, or indeed any alternative formulations of canonical microcircuitry, affect our conclusions is essentially an empirical question that can be addressed through Bayesian model comparison. In other words, the evidence for contrast dependent effects on horizontal (intra and interlaminar) connections may or may not depend upon the details of their deployment or model complexity. The use of more refined models is not limited to neuronal architectures. For example, in this paper we assumed spatially white or uncorrelated inputs to the neural field. Clearly, this is a simplifying assumption that – in principle – can be relaxed by parameterising the spatial spectra or auto covariance functions of the input. This is an important aspect of neural mass modelling of complex cross spectra in the time domain (see Friston et al 2012). We hope to explore this in future studies to see how detailed the models can be, before they become too complex for the data in hand.

The results presented in this paper are consistent with recent non-invasive human studies of contrast dependent changes in the gain of pyramidal cells elaborating forward connections — studies using dynamic causal modelling to test specific hypotheses about precision and gain control based on predictive coding. Furthermore, they are consistent with intriguing results showing that the receptive fields of V1 units shrinks with increasing visual contrast. Methodologically, this work speaks to the potential usefulness of biologically informed generative models of empirical electrophysiological data; especially in answering questions about the functional architectures that underlie neuronal computations.

## Figures and Tables

**Table 1:** Prior expectations of model parameters. (The spatial parameters assume the cortical patch has a diameter of ℓ = 25 mm).

Parameter	Physiological interpretation	Prior mean
*κ*_1_, *κ*_2_, *κ*_3_, *κ*_4_	Postsynaptic rate constants	1/2, 1/35, 1/35, 1/2 (ms^− 1^)
*α*_11_,*α*_14_,*α*_12_ *α*_22_,*α*_21_,*α*_23_,*α*_33_ *α*_41_,*α*_32_,*α*_44_	Amplitude of intrinsic connectivity kernels (× 10^3^)	108,45,1.8 9,162,18,45 36,18,9
*c_ab_*	Spatial decay of connectivity kernels	$\left\{\begin{array}{cc} 0.6 & a\neq b\\ 2 & a=b \end{array}\right)$ (mm^− 1^)
*r*,*η*	Parameters of the postsynaptic firing rate function	0.54, 0
*s*	Conduction speed	.3 m/s
*ϕ* *q*_1_,*q*_2_,*q*_3_,*q*_4_	Dispersion of the lead field Neuronal contribution weights	$\sqrt{2}/16$ .2,0,.2,.6

## Footnotes

2 Not to be confused with the neuronal weights *Q* = *q*_1_,*q*_2_,*q*_3_,*q*_4_.
